# Medications and Orthodontic Tooth Movement: What Accelerates and Diminishes Tooth Movement?

**DOI:** 10.7759/cureus.61840

**Published:** 2024-06-06

**Authors:** Rawan Alrehaili, Ashraf Alhujaili, Shahad Alharbi, Lamia Alharbi, Wejdan Alharbi, Raghad Alkhattabi, Danah Alkhateeb, Rema Albisher, Areej Hakami, Ahmed Khalil

**Affiliations:** 1 Dentistry, Private Practice, Medina, SAU; 2 Dentistry, Primary Health Care, Medina, SAU; 3 Dentistry, University of Hail, Hail, SAU; 4 Dentistry, Vision Colleges, Riyadh, SAU; 5 Dentistry, Princess Nourah Bint Abdulrahman University, Riyadh, SAU; 6 Dentistry, University of Manchester, Manchester, GBR; 7 Dentistry, Private Practice, Buraydah, SAU; 8 Dentistry, Private Practice, Tabuk, SAU; 9 Orthodontics, Private Practice, Alexandria, EGY

**Keywords:** bone remodeling, otm, orthodontic tooth movement, drug, medication

## Abstract

The biological aspect of orthodontic tooth movement is influenced by the magnitude and duration of the applied force. This initiates signaling cascades essential for bone remodeling, which involve activating various cell signaling pathways that enhance the metabolism of the periodontal ligament, leading to localized bone resorption and deposition. This process facilitates tooth movement on the pressure side and promotes healing on the tension side. The remodeling associated with orthodontic tooth movement is an inflammatory reaction involving mediators. Key components in this process include hormones, systemic influences, cyclic adenosine monophosphate, specific cytokines like interleukin 1, colony-stimulating factors, calcium, collagenase, and prostaglandins, all of which are essential for the biological adjustments necessary for tooth movement. Medications that influence molecular pathways critical for the homeostasis of periodontal tissues or that affect changes during orthodontic tooth movement and clastic cell regulation can potentially modulate tooth movement. With the recent increase in prescription medication use, it is essential for clinicians to be aware of medication consumption in prospective patients and understand its potential impact on orthodontic treatment. This review aimed to explore the effects of commonly prescribed medications on the rate of orthodontic tooth movement, thoroughly review the existing evidence on this topic, and identify potential areas for future research.

## Introduction and background

Orthodontic tooth movement is a process where applied force causes bone resorption on the pressure side and bone formation on the tension side. It was described by Proffit et al. as the biological response triggered by disrupting the physiological balance of the dentofacial complex using an externally applied force [1]. The mechanics of tooth movement adhere to Newton's laws while the biological aspects are influenced by the magnitude and duration of the applied force and the resulting signaling cascades that promote bone remodeling. Bone remodeling is a crucial component of orthodontic tooth movement [2]. It involves the activation of various cell signaling pathways that enhance the metabolism of the periodontal ligament leading to localized bone resorption on the pressure side and bone deposition on the tension side [3,4].

The remodeling associated with orthodontic tooth movement is also recognized as an inflammatory reaction. This inflammation within the periodontal ligament space subsequently prompts the release of various biochemical signals and mediators [5]. These are crucial for the remodeling of the alveolar bone and periodontal ligament, which in turn cause tooth movement. In addition, inflammatory mediators, neurotransmitters, and growth factors are critical to this process [5]. The primary elements involved in this dynamic process include hormones, systemic influences, cyclic adenosine monophosphate (cAMP), specific cytokines such as interleukin 1, colony-stimulating factors, calcium, collagenase, and prostaglandins, all of which are essential in facilitating the necessary biological remodeling for tooth movement [6]. The classical theory of tooth movement posits that applying force generates differential pressure in the periodontal ligament, with this effect localized to the endosteal marrow spaces and the cells found in the ligament itself [7]. Medications can significantly influence these molecular pathways, impacting the homeostasis of periodontal tissues [8-10]. Given the recent rise in prescription medication use, it is crucial for clinicians to identify medication consumption in prospective patients and understand its potential impact on orthodontic treatments [11]. Prescription drugs are typically associated with adults, who make up over a quarter of the orthodontic demographic, and their consumption among school-aged children is also on the rise [12-14]. Additionally, the use of over-the-counter medications has continued to increase [15]. This narrative review aimed to revisit the impact of commonly prescribed medications on the rate of orthodontic tooth movement, thoroughly review the existing evidence on this topic, and pinpoint potential avenues for future research.

## Review

Search strategy

A thorough literature search was performed across multiple recognized electronic databases, including PubMed, Embase, MEDLINE, and Google Scholar, to identify peer-reviewed articles relevant to the review up to February 1, 2024. The search terms used included the following: (drug* OR medication OR pharmaceutical OR analgesics OR non-steroidal anti-inflammatory drugs OR bisphosphonate OR relaxin OR fluoride OR hormone OR vitamin OR NSAID OR acetaminophen OR corticosteroid OR statin OR dietary calcium OR vitamin D OR thyroxine OR insulin) AND (tooth movement OR teeth movement OR orthodontic* movement); (tooth or teeth or orthodontic*) and move* and (drug* or medic*); (tooth or orthodontic) and movement and (drug or medication); (drug or medication) and (tooth or orthodontic*) and movement.

Inclusion criteria included original research articles, review articles, systematic reviews, clinical trials, and animal studies. The search was limited to articles published in English. The screening process involved initial title and abstract screening to determine relevance. Articles that met the initial criteria underwent a full-text review to ensure they were pertinent to the review topic. Articles not published in English, studies that addressed unrelated aspects of orthodontic tooth movement, opinion pieces, editorials, and case reports with limited generalizability were excluded.

Nonsteroidal anti-inflammatory drugs

Nonsteroidal anti-inflammatory drugs (NSAIDs) effectively alleviate pain without affecting consciousness by targeting both the central and peripheral pain pathways [16]. In orthodontics, NSAIDs are frequently used to manage pain and offer analgesic, antipyretic, and anti-inflammatory effects [17]. NSAIDs work by blocking the cyclooxygenase enzymes (COX-1 and COX-2) in the arachidonic acid pathway, which suppresses the production of all prostanoids, including thromboxanes, prostacyclins, and prostaglandins [18]. Notably, prostaglandins (PGE1 and PGE2) play a critical role in bone resorption, and their inhibition by NSAIDs might affect the rate of tooth movement due to changes in vascular and extravascular matrix remodeling [19]. Studies exploring the impact of NSAIDs on orthodontic treatment revealed an appreciable effect on tooth movement. Knop et al. [20] found that NSAIDs such as ibuprofen, indomethacin, and acetylsalicylic acid reduce osteoclast-like cells and inhibit tooth movement during the initial stages of treatment, likely due to their effect on prostaglandin synthesis, which plays a crucial role in osteoclastic activity. In the same vein, Retamoso et al. [21] observed that dexamethasone slows down collagen maturation in the periodontal ligament. On the other hand, Consolaro et al. [22] reviewed various studies on the prescription of analgesics after the application of orthodontic force and noted inconsistencies in findings, which complicate direct comparisons. This was due to variations in orthodontic appliances, drug dosages, durations, and administration routes. Further illustrating these dynamics, Yamasaki et al. [23] investigated the effects of indomethacin, a specific inhibitor of thromboxane A2 synthesis on rats and noted a similar inhibition of osteoclasts in the bone's interradicular septum. In essence, Arias and Marquez-Orozco's [24] study on ibuprofen also demonstrated a significant decrease in orthodontic tooth movement, attributing this to the drug’s potent anti-inflammatory properties and its inhibition of prostaglandin synthesis.

These results highlighted the potential for NSAIDs to diminish osteoclast numbers and thus decrease the rate of orthodontic tooth movement.

Acetaminophen

The relationship between acetaminophen and orthodontic tooth movement has been extensively explored across various studies. Once again, NSAIDs inhibit prostaglandin synthesis both centrally and peripherally. However, acetaminophen targets prostaglandin synthesis primarily in the central nervous system and does not function as an anti-inflammatory in peripheral tissues [25]. Several studies consistently found that ibuprofen and aspirin resulted in decreased tooth movement compared to acetaminophen, which maintained movement rates similar to the control group [26,27]. This was further highlighted by Arias and Marquez-Orozco [24], who examined both the pharmacological effects and the histological changes in bone resorption under orthodontic forces, showing that aspirin and ibuprofen significantly decreased osteoclast activity and decreased orthodontic tooth movement, unlike acetaminophen. Bird et al. [28] described similar clinical effectiveness of acetaminophen and ibuprofen when administered preoperatively, suggesting that acetaminophen might be equally effective for initial pain management in orthodontic procedures. In the same vein, Bradley et al. [29] claimed that acetaminophen provided comparable pain relief. However, it did not perform as ibuprofen. This indicated that although acetaminophen can be an effective analgesic for pain, varying dosages or additional doses might be necessary to manage prolonged discomfort with orthodontic procedures [30].

Assessing pain relief options: nonsteroidal anti-inflammatory drugs vs. acetaminophen in orthodontics

**Figure 1:**
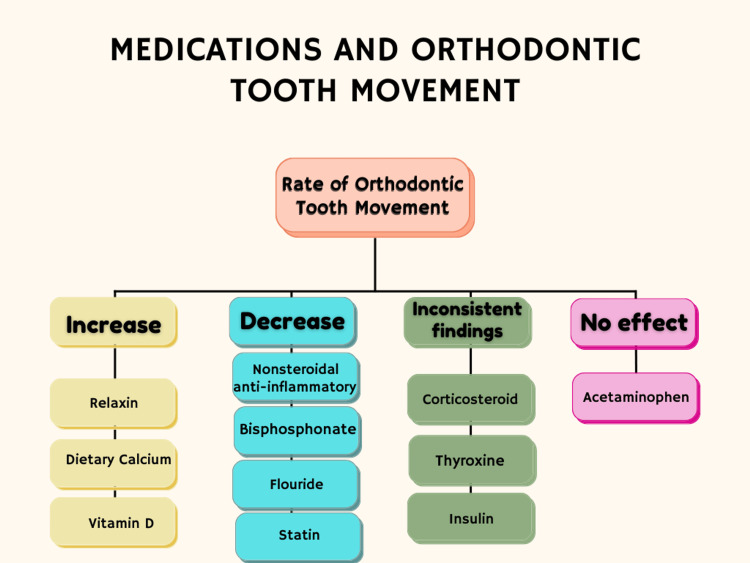
Summary of the effect of the included medications on the rate of orthodontic tooth movement. Image credits: Rawan Alrehaili.

The absence of a detrimental impact on tooth movement makes acetaminophen a feasible option for managing orthodontic pain. For this reason, several researchers have advocated for acetaminophen as the preferred analgesic during orthodontic procedures [24,26,27]. However, because acetaminophen lacks anti-inflammatory properties and the pain associated with orthodontic adjustments is largely inflammatory, its effectiveness in relieving pain compared to NSAIDs is still debatable [31]. Currently, there is no definitive protocol for managing orthodontic discomfort. Considering the possibility that ibuprofen may slow tooth movement without offering superior pain relief compared to acetaminophen, it seems wise for orthodontists to recommend acetaminophen as the preferred medication for pain management in orthodontic care. Although multiple research studies have been conducted on various analgesics and dosing schedules, the results regarding their effectiveness remain debatable. The effect of the included medications on orthodontic tooth movement is summarized in Figure 1.

Bisphosphonate

Bisphosphonates are commonly prescribed to manage bone diseases such as osteoporosis and metastatic bone disease by inhibiting osteoclast-mediated bone resorption. This pharmacological action, while beneficial for increasing bone density and reducing fractures, can complicate orthodontic treatments that rely on bone remodeling to facilitate tooth movement. Bisphosphonates impair osteoclast activity, which in turn leads to a slower rate of tooth movement and potentially longer treatment durations. Research has demonstrated that bisphosphonates, such as alendronate, can decrease the number of osteoclasts available to resorb bone on the pressure side of a moving tooth, which eventually diminishes the orthodontic process [32]. Bisphosphonates, distinguished by their non-amino and amino subclasses based on side chains attached to the central carbon, significantly influence osteoclast function [33]. Non-amino bisphosphonates reduce osteoclast activity through the production of toxic adenosine triphosphate metabolites, whereas amino bisphosphonates disrupt cytoskeletal function and intracellular signaling by inhibiting the enzyme farnesyl pyrophosphate synthase within the mevalonate pathway, leading to osteoclast apoptosis [34].

The effects of bisphosphonates on orthodontic tooth movement and relapse have been explored in various studies. Kim et al. [32] reported that systemic administration of pamidronate in rats resulted in notable structural changes in osteoclasts, such as loss of ruffled borders and cytoplasmic polarity, thereby reducing orthodontic relapse. Similarly, Adachi et al. [35] investigated the dose-dependent effects of topically administered risedronate on orthodontic tooth movement in rats, finding significantly reduced movement. Further research by Karras et al. [36] examined the impact of alendronate, a commonly prescribed bisphosphonate for post-menopausal osteoporosis, on orthodontic tooth movement in rats. Results indicated a significant reduction in tooth movement with alendronate treatment, highlighting its potent inhibitory effects on bone remodeling activity. However, the use of bisphosphonates raises several concerns, particularly regarding growth effects, the risk of bisphosphonate-associated osteonecrosis of the jaws, and their long half-life, which could pose residual effects years after administration [37]. These issues reflect the need for cautious application of animal model results to humans. This caution is particularly important given the severe complications like osteonecrosis, which can lead to significant morbidity, including bone sequestration and pathological fractures [38].

Considering these complexities, it becomes imperative to approach bisphosphonate use in orthodontics with careful consideration of both their profound inhibitory effects on bone resorption and the potential risks associated with long-term use and specific health conditions.

Corticosteroids

In a series of studies examining the effects of corticosteroids on orthodontic tooth movement, different outcomes were observed based on the type of corticosteroid and the experimental setup. Research involving rabbits treated with cortisone acetate for four days prior to and during the application of an orthodontic force demonstrated a significant acceleration in orthodontic tooth movement [39]. Interestingly, this group also experienced a quicker relapse rate compared to controls. Further studies on prednisolone provided insights into dose-dependent effects. When administered through drinking water over a 14-day period to rats, a suppression of orthodontic tooth movement was observed [40]. However, another experiment using a higher dose for a 24-day period split into induction and active movement phases showed no significant impact on the rate of orthodontic tooth movement [41]. Studies on methylprednisolone also highlighted variable effects depending on the protocol used. In one experimental group, a seven-week induction led to an increase in the rate of orthodontic tooth movement [42]. Conversely, another group without an induction period but receiving the same treatment did not show any change in orthodontic tooth movement rates. In addition, triamcinolone, particularly its derivative triamcinolone acetonide, was investigated in rabbits where it was injected for 21 days while applying a force to move the incisors over the same period. This resulted in a significant increase in orthodontic tooth movement [43].

Findings collectively suggested that the effects of corticosteroids on orthodontic tooth movement are influenced by several factors, including the specific type of corticosteroid, duration of treatment, and the presence of an induction period. This was supported by the review of Michelogiannakis et al. [44], who concluded that the impact of corticosteroids on orthodontic tooth movement remains uncertain due to the varied and inconsistent methodologies of the studies.

Relaxin

Relaxin, a hormone from the insulin/relaxin family, is initially known for facilitating the widening of pubic ligaments during childbirth, but it also possesses roles in collagen turnover, angiogenesis, and anti-fibrotic effects [45,46]. Within orthodontics, its potential to aid tooth movement is notable. Research by Madan et al. [47] indicated that relaxin promotes orthodontic tooth movement by increasing collagen levels at areas of tension and reducing them at compression sites, thereby supporting fiber and bone remodeling where tension exists. However, findings from McGorray et al. [48] suggested that weekly relaxin injections over an eight-week period did not alter the rate of tooth movement, presenting a conflicting perspective. Additionally, studies involving the local administration of human relaxin in rats demonstrated that while relaxin can speed up orthodontic tooth movement, it may also compromise the organization and integrity of the periodontal ligament [49,50].

Evidence highlighted that while relaxin can enhance certain aspects of orthodontic treatment by affecting soft tissue dynamics and collagen distribution, its comprehensive impact on tooth movement and periodontal ligament condition requires cautious evaluation. The dual nature of relaxin’s effects underscored the need for more in-depth research to elucidate its potential benefits and risks in orthodontic practice.

Fluoride

Fluoride salts served as important chemical reagents and industrial chemicals, predominantly in the production of hydrogen fluoride for fluorocarbons [4]. Fluoride's impact on orthodontic tooth movement has been studied both in animal models and human subjects, revealing complex interactions with bone remodeling processes and hence orthodontic treatment. Early research by Hellsing and Hammarstrom [51] used subcutaneous osmotic pumps in rats to deliver fluoride, which significantly reduced the rate of tooth movement and decreased the number of osteoclasts on the pressure side of the periodontal ligament. This suggested that fluoride might inhibit the bone resorption process, which is vital for tooth realignment. Further exploring fluoride's effects, Gonzales et al. [52] observed that continuous fluoride exposure from birth in rats reduced the rate of tooth movement. In addition, the longer the fluoride exposure, the lesser the tooth movement observed. This was aligned with the finding that high concentrations of fluoride could have inhibitory effects on orthodontic tooth movement by affecting osteoclast activity. Contrastingly, a study conducted by Karadeniz et al. [53] examined the effects of high and low fluoride concentrations in drinking water on orthodontic tooth movement in humans. The authors found that higher fluoride intake when combined with heavy orthodontic forces increased the rate of tooth movement compared to lower fluoride levels. As expected, heavy force combined with high fluoride intake resulted in the greatest average rate of tooth movement. The study also noted a significant negative correlation between age and tooth movement, suggesting that younger individuals may experience more rapid tooth movement.

These findings suggested that fluoride's impact on orthodontic tooth movement can vary, primarily inhibiting movement in many contexts.

Statins

Statins, widely known for their cholesterol-lowering properties, have been investigated for their potential impact on orthodontic tooth movement. The impact of statins on orthodontic tooth movement is primarily due to their modulation of the bone remodeling process, which is a critical component of tooth movement [54]. Several studies have examined the effects of different statins, primarily simvastatin and atorvastatin, in animal models and clinical settings. Experimental studies by Dolci et al. [55] and Esfahani et al. [56] found that systemic administration of simvastatin led to decreased orthodontic tooth movement and inhibited osteoclastogenesis in rats. Similarly, a study reported reduced root resorption and enhanced bone formation in dogs treated with simvastatin [57]. MirHashemi et al. [54] observed that atorvastatin administration in rats resulted in both reduced orthodontic tooth movement and inhibited bone resorption, supporting the notion that statins can modulate bone remodeling processes crucial for orthodontic treatment outcomes. However, not all studies reported consistent results. Vieira et al. [58] found minimal to no inhibition of tooth movement in post-orthodontic release models upon simvastatin administration. Furthermore, the clinical study by Jahanbin et al. [59] noted that while simvastatin could reduce space reopening in orthodontic treatments, its impact on overall orthodontic tooth movement and gingival health varied.

These discrepancies highlighted the variability in statin effects based on dosage, duration, and specific statin used. Despite the decreased rate of tooth movement in most studies, there remains a need for further research to clarify the conditions under which statins might be beneficial or detrimental to orthodontic treatment, considering the high risk of bias and methodological inconsistencies across studies.

Dietary calcium

Dietary recommendations for calcium intake vary by age, with children aged four to eight years advised to consume 800 mg/day and adults recommended between 1000 and 1300 mg/day [60]. Typically, the net absorption of calcium from diet is only about 500 mg/day. While calcium supplementation is frequently prescribed to prevent osteoporosis in postmenopausal women, its routine use has been questioned due to potential cardiovascular risks [61]. The impact of dietary calcium on orthodontic tooth movement has been investigated in several animal studies, which yielded consistent findings. In a study conducted on dogs, the animals were placed on either low- or high-calcium diets for a period of 10 weeks. The low-calcium diet resulted in a significantly higher rate of orthodontic tooth movement compared to the high-calcium diet [62]. The mechanism behind this could be due to the increased bone resorption, altered hormonal regulation, and disrupted balance of bone remodeling cells and inflammatory mediators [62]. These findings aligned with a similar study in rats where lactating animals were fed a low-calcium diet for one week [63]. The rate of orthodontic tooth movement in these rats was higher than in the control group. This increased rate of tooth movement in low-calcium conditions was likely due to the rapid response of trabecular bone to reduced calcium intake, which triggered an increase in parathyroid hormone release and subsequently stimulated bone remodeling.

These studies reflected the role of dietary calcium in influencing the rate of orthodontic tooth movement through its effects on bone metabolism and remodeling processes.

Vitamin D

Calcium and phosphorus levels are controlled by vitamin D, parathyroid hormone, and calcitonin. Vitamin D, particularly in its active form, 1,25-dihydroxycholecalciferol, is a powerful activator of osteoclast precursors, affecting both osteoclasts and osteoblasts [11]. It was claimed that the local administration of calcitriol can enhance tooth movement in humans in a dose-dependent manner [64]. Collins and Sinclair [65] found that weekly intra-ligamentous injections of a vitamin D metabolite significantly increased the number of osteoclasts and enhanced tooth movement during light-force canine retraction over a 21-day experimental period. In the same line, it was observed that rats receiving vitamin D treatment exhibited increased bone formation on the pressure side of the periodontal ligament following the application of orthodontic forces [11]. Similarly, Kale et al. [66] noted that vitamin D promotes balanced bone turnover, thereby accelerating tooth movement in rats. Kawakami et al. [67] reported a higher rate of mineral deposition in alveolar bone following orthodontic force application, suggesting that the local application of vitamin D could enhance the recovery of supporting alveolar bone after orthodontic treatment.

These findings determined the potential of vitamin D as a therapeutic agent in optimizing orthodontic outcomes through its influence on bone remodeling and turnover.

Thyroxine

The influence of thyroxine on orthodontic tooth movement revealed inconsistent results across the studies. Seifi et al. [68] found that thyroxine administration notably increased the rate of orthodontic tooth movement in rats. This finding was supported by the two research studies performed by Verna et al. [69,70], who also reported enhanced tooth movement with thyroxine supplementation. Conversely, Baysal et al. [71] observed no significant effect of thyroxine on tooth movement, a result consistent with those of Poumpros et al. [72] and Jung et al. [73]. Shirazi et al. [61] provided an interesting nuance by showing that a higher dose of thyroxine (20 µg/kg) increased the rate of orthodontic tooth movement, whereas lower doses (5 and 10 µg/kg) did not produce significant changes. These variations in findings can be attributed to differences in study designs, including dosage, administration methods, and experimental models. The inconsistent results highlight the necessity for more standardized research to determine the precise role of thyroxine in orthodontic treatments. This was further supported by the systematic review of Berry et al. [74], who concluded that the relationship between thyroxine administration and orthodontic tooth movement remains inconclusive.

Insulin

In the study by Braga et al. [75], accelerated orthodontic tooth movement was observed in diabetic mice. This was attributed to an increased osteoclast count suggesting that diabetes mellitus induces orthodontic tooth movement through the proliferation of osteoclasts. Contrarily, Arita et al. [76] found that diabetes mellitus reduces bone resorption, resulting in a decreased rate of orthodontic tooth movement. However, this study did not examine the effect of diabetes mellitus on bone cells or periodontal biomarkers, leaving the exact cause of diminished orthodontic tooth movement unclear. In the same vein, Villarino et al. [77] demonstrated that insulin administration mitigated the effects of diabetes mellitus and increased the rate of orthodontic tooth movement to levels comparable to non-diabetic mice. This suggested that well-controlled blood glucose levels in diabetic patients may minimize the impact of diabetes mellitus on orthodontic tooth movement. While the administration of insulin showed conflicting results, it generally normalized the rate of orthodontic tooth movement to that of normoglycemic subjects [75,76]. Nevertheless, a systematic review by Najeeb et al. [78] concluded that insulin might adversely affect bone remodeling and orthodontic tooth movement. Furthermore, it stressed the need for research to further explore the impact of insulin on orthodontic tooth movement.

A summary of the included medications and their effect on orthodontic tooth movement with the involved mechanism is depicted in Table 1.

**Table 1:** Summary of the included medications and their effect on orthodontic tooth movement with the involved mechanism.

Medication	Role	Effect on orthodontic tooth movement and mechanism
Nonsteroidal anti-inflammatory drugs (NSAIDs)	Analgesic, anti-inflammatory and antipyretic	Decreases orthodontic tooth movement by reducing osteoclast activity and inhibiting prostaglandin synthesis
Acetaminophen	Analgesic	Maintains normal orthodontic tooth movement rates similar to control groups
Bisphosphonate	Management of bone diseases	Decreases orthodontic tooth movement by reducing osteoclast numbers and impairing bone resorption
Corticosteroid	Anti-inflammatory	Inconsistent findings
Relaxin	Hormone facilitating collagen turnover	Increases orthodontic tooth movement by increasing collagen levels at tension sites and reducing them at compression sites
Fluoride	The production of hydrogen fluoride for fluorocarbons	Inhibits orthodontic tooth movement by reducing osteoclast activity
Statin	Cholesterol-lowering	Decreases orthodontic tooth movement by inhibiting osteoclastogenesis and enhancing bone formation, though effects can vary based on specific statin and dosage
Dietary calcium	Bone health	Increases orthodontic tooth movement due to increased bone resorption
Vitamin D	Regulates calcium and phosphorus	Increases orthodontic tooth movement by promoting osteoclast activity and bone turnover
Thyroxine	Metabolic rate regulator	Inconsistent findings
Insulin	Blood glucose regulation	Inconsistent findings

Limitations

The current review has several limitations primarily due to the nature and characteristics of the included studies. Many studies exhibited insufficient data on blinding and reliability of measurement methods for assessing tooth movement. Moreover, most of the findings from this review were derived from animal studies, limiting their direct applicability to human scenarios. The substances were administered over short periods, unlike the extended durations often seen with prescribed medications, at dosages and via routes that differ from those typically used in human clinical settings. Additionally, the focus on specific biomechanical systems in the studies limits the generalizability of the findings to human clinical practice. Furthermore, most studies lacked power calculations, adding another layer of limitation regarding the precision of the findings. Consequently, it is somewhat unclear which medications may have a clinically significant impact on the treatment outcomes in real-world clinical settings.

Recommendations for future research

The recent increase in the use of both prescribed and non-prescribed medications across various age groups highlighted the need for more rigorously designed experimental and, where feasible, clinical trials on how these substances affect orthodontic tooth movement. Standardizing research methodologies is essential, along with carefully addressing potential bias risks. Furthermore, it is important to carefully choose study parameters such as duration, dosage, and method of administration, as well as the details of the biomechanical systems used, to closely replicate clinical conditions in humans. However, the challenge of ethical concerns regarding the implementation of a drug on humans without sufficient animal trials must be acknowledged.

## Conclusions

The growing exposure of individuals across all age groups to a wide variety of medications highlights the crucial role of a detailed medical history in orthodontic practice. It is essential for orthodontists to meticulously assess the medication history of the patients, both before initiating and during the course of orthodontic treatment. This thorough evaluation is the key to tailoring treatment plans effectively, allowing for precise adjustments in force application and appointment scheduling. Moreover, clinicians must be proficient in identifying patients who are taking medications and thoughtfully consider how these drugs impact the overall treatment strategy. This comprehensive approach ensures that orthodontic care is both safe and effective, accommodating the unique needs of each patient.
